# West Nile Virus Range Expansion into British Columbia

**DOI:** 10.3201/eid1608.100483

**Published:** 2010-08

**Authors:** David Roth, Bonnie Henry, Sunny Mak, Mieke Fraser, Marsha Taylor, Min Li, Ken Cooper, Allen Furnell, Quantine Wong, Muhammad Morshed, Members of the British Columbia West Nile Virus Surveillance Team

**Affiliations:** British Columbia Centre for Disease Control, Vancouver, British Columbia, Canada (D. Roth, B. Henry, S. Mak, M. Fraser, M. Taylor, M. Li, K. Cooper, A. Furnell, Q. Wong, M. Morshed; University of British Columbia, Vancouver (D. Roth, B. Henry, M. Morshed); 1 Members of the Surveillance Team are listed at the end of this article.

**Keywords:** West Nile virus, arbovirus, Canada, British Columbia, insect vectors, arthropod vectors, ecology, zoonoses, epidemiology, research

## Abstract

In 2009, an expansion of West Nile virus (WNV) into the Canadian province of British Columbia was detected. Two locally acquired cases of infection in humans and 3 cases of infection in horses were detected by ELISA and plaque-reduction neutralization tests. Ten positive mosquito pools were detected by reverse transcription PCR. Most WNV activity in British Columbia in 2009 occurred in the hot and dry southern Okanagan Valley. Virus establishment and amplification in this region was likely facilitated by above average nightly temperatures and a rapid accumulation of degree-days in late summer. Estimated exposure dates for humans and initial detection of WNV-positive mosquitoes occurred concurrently with a late summer increase in *Culex tarsalis* mosquitoes (which spread western equine encephalitis) in the southern Okanagan Valley. The conditions present during this range expansion suggest that temperature and *Cx. tarsalis* mosquito abundance may be limiting factors for WNV transmission in this portion of the Pacific Northwest.

## Abstract 2

Elevated temperatures and mosquito abundance may contribute.

West Nile virus (WNV) is a vector-borne flavivirus that is transmitted in an enzootic cycle between birds by mosquitoes; incidental transmission to humans occurs during periods of intense amplification, typically in late summer in the Northern Hemisphere (*1*). WNV activity is inherently dependent on environmental and ecologic conditions that affect avian and vector populations because of the role these groups play in WNV transmission (*2*). Environmental factors such as temperature (*3**,**4*), precipitation (*4*), and drought (*5*), and ecologic conditions such as vector abundance (*6*) have been identified as possible determinants of WNV activity.

Canada represents the northernmost range of WNV in North America. The first positive WNV indicators appeared in Canada in 2001 when the virus was detected in birds and mosquitoes in Ontario (*7*). A total of 394 human cases occurred in Ontario and 20 in Quebec during 2002 (*7*). The virus quickly spread westward into the prairie provinces: 947 confirmed cases in Saskatchewan in 2003, of which 63 were West Nile neurologic syndrome (WNNS) (*8*), 144 in Manitoba (35 WNNS) (*9*), and 275 (48 WNNS) in Alberta (*10*). A second major outbreak occurred in Canada in 2007, a total of 1,456 (113 WNNS) cases were confirmed in Saskatchewan (*8*), 587 (72 WNNS) in Manitoba (*9*), and 318 (21 WNNS) in Alberta (*10*). Although mostWNV activity has occurred in the southern parts of the country, the virus has been detected as far north as Meadow Lake, Saskatchewan (54°08′N) (*11*).

Despite this widespread activity, no local WNV transmission was detected in Canada’s westernmost province, British Columbia, during the WNV seasons (May to October) of 1999–2008 (*7**,**11*). The absence of WNV in British Columbia during this period puzzled provincial public health experts, who had been preparing for the virus’s arrival since 2002; some speculated that British Columbia did not contain the prerequisite environmental and ecologic conditions essential for WNV activity. However, in August 2009, a long-delayed range expansion of WNV into British Columbia was confirmed; 2 locally acquired cases in humans, 10 positive mosquito pools, and 3 cases in horses were detected by provincial surveillance.

The official arrival of WNV in British Columbia puts to rest the question of whether the province can sustain within-season WNV activity. However, new questions have been raised relating to the mechanism of viral introduction, the environmental conditions that limited previous WNV activity in the province, the focus of WNV activity in the southern Okanagan Valley, and whether British Columbia can sustain activity between seasons. We examined spatial and temporal patterns of WNV activity in British Columbia in relation to mosquito abundance and temperature conditions present during the observed range expansion of 2009. Our goal was to identify potential determinants of WNV activity along this portion of British Columbia’s northern and western ranges and to provide additional information regarding factors that facilitate the spread of WNV in North America.

## Material and Methods

### Study Area

**Figure 1:**
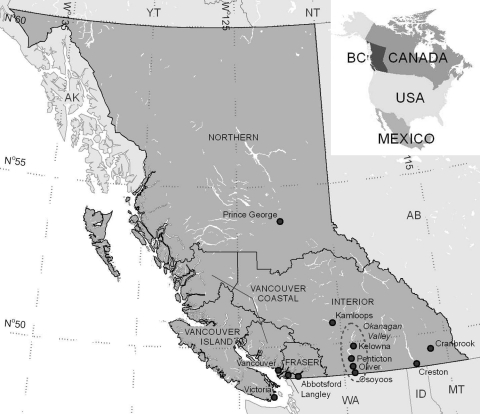
Select cities (lower case) in British Columbia, Canada, and Regional Health Authorities (RHA, upper case). Each RHA undertakes West Nile Virus surveillance under the guidance and recommendations of the British Columbia Centre for Disease Control. The dashed oval encompasses the Okanagan Valley, which was the primary focal point of West Nile Virus activity in British Columbia during 2009. WA, Washington, USA; ID, Idaho, USA; MT, Montana, USA.

The province of British Columbia is an ecologically, climatically, and geomorphologically diverse area covering 947,000 km^2^ that contains a lengthy coastline, high mountain ranges, and a desert region (Figure 1). British Columbia has the most geological, climatic, and biological diversity in Canada (*12*). This province is dominated by vast regions of temperate forests in mountainous areas >1,000 m above sea level (*13*). Temperatures in the coastal regions of British Columbia are among the mildest in Canada; daily average temperatures are above freezing year-round (*14*). The coastal regions receive >1,100 mm of rain per year as moisture-laden air from the Pacific Ocean rises above the Coast Mountain Range, resulting in orographic precipitation. In contrast, the southern interior of the province is part of the semiarid steppe highlands ecoregion, which has near desert-like conditions including hot dry summers, cool winters, and average rainfall of 260 mm per year (*14*).

### Provincial WNV Surveillance

The British Columbia Centre for Disease Control (BCCDC) and the BCCDC Public Health Microbiology and Reference Laboratory (PHMRL), in partnership with regional health authorities, municipalities, and regional governments, have conducted human surveillance, mosquito sampling, and dead corvid surveillance and testing since 2003. During the WNV seasons of 2003–2007, mosquito surveillance covered the southern extent of the province and extended as far north as 55°N latitude. However, in response to the prolonged absence of the virus, mosquito surveillance was reduced in 2008; mosquito traps were placed only at or below 50°N latitude (Table 1; Figure 1). An additional 16 traps were placed in the southern Okanagan Valley in 2009 as part of a research project to supplement the 91 traps operated by the province, effectively acting as targeted surveillance in this area. CDC light traps (Model 512; John W. Hock Company, Gainsville, FL, USA) baited with dry ice were run 1 or 2 nights per week from June through September.

**Table 1:** Summary of British Columbia WNV surveillance activities during the WNV seasons of 2004–2009*

Surveillance type	2004	2005	2006	2007	2008	2009
Mosquito						
No. permanent trap locations	145	189	148	155	98	91
No. mosquitoes collected	52,657	198,228	394,047	242,215	202,460	181,942
Mosquito pools†	2,980	6,631	2,329	2,568	1,873	1,469
Provincial						
No. *Culex pipiens*	4.6	5.1	8.6	14.3	10.5	21.1
No. *Cx. tarsalis*	0.8	1.9	4.8	3.5	1.4	11.8
Interior‡						
No. *Cx. pipiens*	12.6	8.9	9.5	8.2	4.7	7.6 (9.3)
No. *Cx. tarsalis*	11.0	12.0	22.5	10.1	2.9	33.1 (14.3)
Fraser						
No. *Cx. pipiens*	10.5	13.0	22.3	32.5	33.7	36.1
No. *Cx. tarsalis*	6.9	4.9	7.7	7.1	7.0	15.1
Bird						
No. corvids sighted	1,292	740	605	562	458	355
No. corvids submitted	1,437	1,058	803	740	205	144
No. corvids positive	0	0	0	0	0	0
Human						
No. tested	481	755	239	805	530	340
No. positive§	0	0	0	19 (0)	0	3 (2)

**Figure 2:**
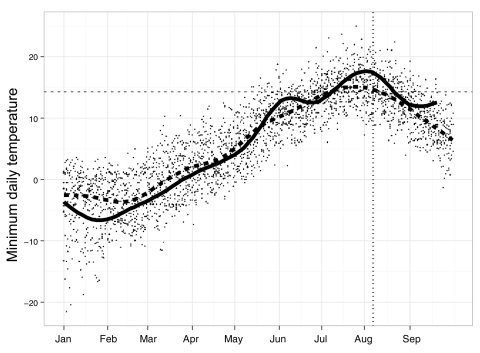
Nightly average catch for *Culex pipiens* (A) and *Cx. tarsalis* (B) mosquitoes across all trapping locations in British Columbia, Canada, during 2005–2009. Provincial vector surveillance data are aggregated by week beginning January 1, and the dates provided represent the first day of a given week. Vertical dashed line represents the estimated exposure date for human cases and the collection date for the first positive mosquito pools.

*WNV, West Nile Virus. †Values represent average trap catch. Provincial, average trap catch for all traps in British Columbia; Interior, average trap catch from the Interior Health Authority, which includes the southern Okanagan Valley (Figure 2); Fraser, average trap catch from the Fraser Health Authority (Figure 2). ‡Values represent average trap catch from all traps run in the southern Okanagan Valley in 2009, including 16 traps run in the area as part of a research project. Values within parentheses represent average trap catch without the research traps. §No. human cases detected in British Columbia (no. cases acquired within British Columbia).

Collected mosquitoes were sent to the BCCDC PHMRL where they were sorted by gender, identified to the genus and/or species level, and pooled to a maximum of 50 mosquitoes per pool. All pools of female *Culex* spp. mosquitoes were homogenized, and RNA was extracted by using a QIAamp Viral RNA Mini Kit (QIAGEN, Valencia, CA, USA). RNA extracts were subjected to an in-house–developed TaqMan real-time reverse transcription–PCR (RT-PCR) screening specific for the 3′ noncoding region and nonstructural protein 5′ of the WNV genome. Positive pools were then confirmed by using a second TaqMan real-time RT-PCR specific for the WNV envelope protein (*15**,**16*).

Passive dead corvid surveillance in British Columbia is conducted by regional health authorities and includes 1) online reporting of dead bird sightings by the public, and/or 2) collection of dead corvids, which are then submitted for testing at the British Columbia Ministry of Agriculture and Lands Animal Health Centre (AHC). Oropharyngeal swabs from dead birds are screened for WNV by using the VecTest (Microgenics Corporation, Fremont, CA, USA); RT-PCR was used as the confirmatory test on pooled tissues from suspected positive birds (*17*).

WNV infection is a reportable disease in British Columbia, and information about probable human cases is communicated to the requesting physician and to public health officials; a case questionnaire is then administered to collect information on symptoms, travel history, and likely mode of transmission. Cases are classified as West Nile nonneurologic syndrome or WNNS according to the case definitions of the Public Health Agency of Canada (*7*). Cases are further categorized as probable or confirmed, depending on the level of specificity associated with the laboratory testing. All potential human case-patients are tested for WNV immunoglobulin M (IgM) and IgG by using ELISA (FOCUS Technologies, Cypress, CA, USA) and acute-phase and convalescent-phase serum samples; in-house hemagglutination inhibition (HI) tests are conducted when needed (*16*). Positive test results from the BCCDC are sent to the National Microbiology Laboratory in Winnipeg, Manitoba, Canada, for confirmatory plaque reduction neutralization testing.

Equine surveillance in British Columbia is passive. Positive equine cases are reported by local veterinarians to the AHC and the provincial chief veterinarian. WNV in horses is identified by using ELISA, serum neutralization, and/or plaque-reduction neutralization test. Horses suspected to have died of WNV are brought to the AHC for diagnostic necropsy. Although equine vaccinations are available in British Columbia, coverage is not widespread with the exception of horses that travel to the United States.

### Temperature Analysis and Degree-Day Calculations

Development of WNV vectors and of the virus within an infected mosquito depends on temperature (*3**,**4*). Degree-day calculations use the product of temperature and time to estimate the cumulative energy required for an organism to develop (*18*). An estimated 109 degree-days are required for the completion of the extrinsic incubation period of WNV in *Cx. tarsalis* mosquitoes; the virus is unable to develop in this species at temperatures <14.3°C (*3*). We used the single-sign method (*19*) with a 14.3°C base to calculate the accumulated degree-days between January 1 and August 1 during 2003–2009 for select British Columbia communities*.* This method combines a 24-h sine wave with daily minimum and maximum thresholds to calculate the accumulated degree-days over 24 hours. The single-sine method provides the most accurate degree-day quantification when daily temperatures are below the minimum development threshold (*20*), and has been used in other WNV studies to estimate risk (*21*). Daily minimum temperature was evaluated for the southern Okanagan community of Osoyoos because it was the closest center to the focal point of WNV activity in British Columbia that also contains an official weather station. The daily minimum temperature for 2009 was compared with the 10-year average by using data from the Canadian National Weather Service, Environment Canada (*14*).

## Results

### Provincial and Regional WNV Activity

In early August 2009, serum samples from 2 residents of Kelowna (49°55′N, 119°30′W) were positive for WNV (Table 1; Figure 1). Travel histories indicated that neither person had been outside of interior British Columbia during the period of potential exposure and that each had recently traveled in the southern Okanagan Valley, which is 70–80 km south of Kelowna (Figure 1). During the same week, provincial surveillance detected a positive mosquito pool; 9 more were detected over the subsequent 2 weeks. All positive pools came from the southern Okanagan Valley and were located up to 35 km apart. Three WNV-positive horses were reported to the chief veterinarian and the AHC in early September: 2 from the southern Okanagan Valley and 1 from the more eastern Fraser Valley (Figure 1). None of the horses had traveled during their exposure period.

With the exception of British Columbia, WNV activity in Canada in 2009 (only 8 human cases nationwide) was among the lowest recorded (*7*). Washington, however, had its greatest WNV activity on record in 2009 (38 cases in humans and 73 cases in horses), up from previous highs of 3 cases in humans and 41 cases in horses or other mammals in 2008 (*22*).

### Mosquito Abundance and Infection Rates

A total of 181,942 mosquitoes were collected in 2009 from 107 traps (Table 1). The most common mosquitoes in British Columbia are *Coquillettidia* *purturbans* and members of the genus *Aedes*. British Columbia is the only area in western Canada that has *Cx*. *tarsali*s and *Cx*. *pipiens* mosquitoes; the former are rare east of the Mississippi River, and the latter are absent in the prairie provinces (*23*). However, the abundance of these species is typically lower than in the prairie provinces of Saskatchewan and Manitoba, which experience the most intense WNV transmission in Canada (*8**,**9*). *Cx*. *pipiens* mosquito abundance in the Fraser Valley in 2009 increased relative to previous years; an average of 36.1 mosquitoes were caught per trap-night. An average of 33.1 *Cx. tarsalis* mosquitoes were caught per trap-night in the provincial interior, which was the highest abundance of this species reported in the previous 5 years (Table 1). This average from the southern Okanagan Valley includes data from 16 novel traps placed as part of a targeted research project. However, the average provincial count was still the highest since 2006 when these traps were excluded (Table 1). Peaks in the abundance of *Cx.* *tarsalis* mosquitoes have been observed previously in British Columbia in late June, but a second substantial increase in the abundance of this species was observed in early August 2009. Several locations in the southern Okanagan Valley showed maximum nightly trap counts >800 *Cx. tarsalis* mosquitoes (Figure 2). The first WNV-positive mosquito pools were collected during this period of elevated *Cx. tarsalis* mosquito abundance; the estimated exposure period for both human cases also occurred at this time (Figure 2). *Cx*. *pipiens* mosquitoes consistently increased in the Fraser region throughout the summer; some traps caught up to 750 mosquitoes in a single night in 2009. Although the average trap catch of this species has been increasing continuously in this area since 2003, the abundance of WNV vectors in British Columbia remains much lower than in areas of Canada that have experienced large WNV outbreaks (*8**,**9*).

*Cx*. *tarsalis* was the only vector species in British Columbia that was positive for WNV in 2009. However, only *Cx. tarsalis* and *Cx. pipiens* mosquitoes are tested for WNV in British Columbia. Bias-corrected maximum-likelihood estimates (MLEs) of vector infection rates were calculated by using the Centers for Disease Control and Prevention’s (CDC’s) PooledInfRate Microsoft Excel add-on (*24*). The virus reached detectable levels in late July and peaked in the latter half of August (Table 2) with 2-week MLEs of mosquito infection rates reaching 4.97/1,000 (95% confidence interval [CI] 0.89–16.63) for the weeks of August 23, 2009, to Sepember 5, 2009 (Table 2). Low mosquito abundance has, however, limited the precision of these estimates. Minimum infection rates are larger than comparable MLEs from July 26–August 8 and smaller than MLEs thereafter (Table 2), indicating that >1 infected mosquito may be present per pool as is common when infection rates are high (*24*).

**Table 2:** MLEs and MIRs of 2-week infection rate in *Culex* *tarsalis* mosquitoes, South Okanagan Valley, British Columbia, 2009*

Week	Total no. individuals	No. pools	No. positive pools	MLE (95% CI)	MIR
Jun 28–Jul 11	1,542	52	0	0.00	0.00
Jul 12–Jul 25	670	30	0	0.00	0.00
Jul 26–Aug 8	3,376	98	4	1.21 (0.39–2.92)	1.85
Aug 9–Aug 22	959	67	4	4.49 (1.45–10.95)	4.17
Aug 23–Sep 5	424	37	2	4.97 (0.89–16.63)	4.71
Sep 6–Sep 19	4	1	0	0.00	0.00

*MLE, maximum-likelihood estimate; MIR, minimum infectious rate estimate; CI, confidence interval. Estimates are calculated by using the software PooledInfRate from Centers for Disease Control and Prevention (*24*) and represent number of positive mosquitoes per 1,000 tested. Values within parentheses represent 95% confidence intervals.

### Bird Surveillance

A total of 6,681 corvids were tested for WNV during 2003–2009; none were positive (Table 1). The decreasing number of dead corvids reported since 2006 likely results from a combination of changes to regional surveillance strategies, decreases in the frequency of education campaigns, and changing public perception given the prolonged absence of the virus. We do not believe that the observed decrease resulted from a die-off of WNV-infected birds.

### Climate

**Figure 3:**
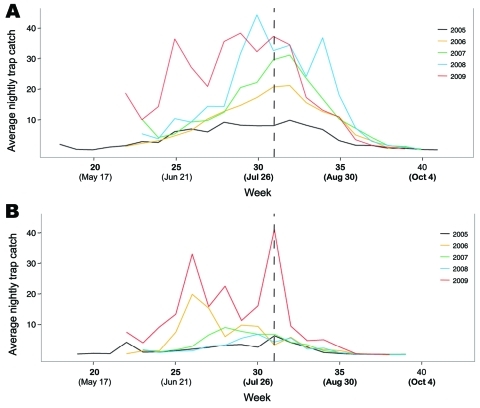
Minimum daily temperature for Osoyoos, British Columbia, Canada, January–September 2009. The solid line represents values observed in 2009; the dashed line represents the best-fit 10-year average. The horizontal dotted line at 14.3°C represents estimated temperature required for *Culex tarsalis* mosquito development and transmission (*3*). The vertical dashed line represents the estimated exposure date for human cases and the collection date for the first positive mosquito pools

More degree-days were accumulated in 2009 for most locations in the province, including Osoyoos, than in any year since 2003–2004 (Table 3). Daily minimum temperatures in the winter and spring in Osoyoos in 2009 were below the 10-year average yet quickly increased in late May and early June and remained above the 10-year-average for much of the summer (Figure 3). The average minimum temperature in July 2009 in Osoyoos (15.5°C) was nearly a full degree higher than the 20-year average; average minimum temperatures in August (15.3°C) were the highest seen in 20 years (*14*). Maximum temperatures reached 34.9°C, 38.6°C, and 39.5°C in June, July, and August, respectively (*14*).

**Table 3:** Cumulative degree-days for communities in British Columbia, January 1–August 31, 2003–2009*

Year	Community							
	Cranbrook	Creston	Osoyoos	Kamloops	Abbotsford	Vancouver	Victoria	Prince George
2003	599	770	962	820	485	408	375	283
2004	479	668	993	880	562	485	425	361
2005	409	581	850	738	481	386	357	275
2006	542	700	851	821	469	366	347	333
2007	561	757	859	738	417	344	311	273
2008	475	611	811	729	387	312	202	265
2009	477	661	919	860	518	422	365	343

*Degree-days are calculated by using the single-sine method (*19*) with a 14.3°C base (3). See Figure 1 for location of select communities.

## Discussion

The delayed establishment of WNV in British Columbia may stem from 1) limited or failed introduction of the virus from adjacent areas with WNV activity before 2009, and/or 2) previously unsuitable environmental or ecologic conditions that prevented establishment, persistence, and amplification of WNV to detectable levels. Although both factors have contributed to the delayed establishment of WNV in British Columbia, they should be clearly separated because virus introduction and persistence are unique events (*25*). The comparative role of these processes in explaining the prolonged absence of WNV in British Columbia is difficult to determine, but the timing and location of British Columbia’s initial WNV activity do provide clues as to potential drivers of this range expansion.

WNV activity in British Columbia in 2009 was centered in the south-central part of the province in the southern Okanagan Valley (Figure 1). Provincial risk maps created by the BCCDC identified this area as having relatively high WNV risk for reasons other than its proximity to the United States. WNV activity is negatively associated with mountainous landscapes (*26*), and the Okanagan Valley is one of the few nonmountainous areas in southern British Columbia. Valleys may act as paths of least resistance for local vector- or reservoir-mediated introduction of the virus into British Columbia from Washington (*27**,**28*) or by migrating birds along the Pacific Flyway (*29*). The southern Okanagan Valley also contains abundant irrigated landscapes that are clustered along with human habitation near the rivers and lakes in the southern Okanagan Valley. This aggregation of favorable habitats brings vectors, reservoirs, and humans into close proximity and may facilitate virus amplification and transmission to humans and horses (*30*).

Not only does the southern Okanagan Valley contain favorable habitats, but it also has a climate that, unlike much of British Columbia, is favorable for WNV amplification and transmission. The southern Okanagan Valley is the hottest region in British Columbia during the summer months. Temperature is positively related to mosquito development rates and the frequency with which mosquitoes take blood meals (*31*). Rates of virus development within mosquito vectors also increase with temperature, and such relationships have consequences for disease transmission because failure of the virus to replicate before mosquito death can halt virus amplification (*3*).

Temperatures in 2009 were above average for much of British Columbia, including the southern Okanagan Valley. WNV outbreaks in the United States and Canada have occurred primarily during years with above-normal temperatures (*7**,**32*), and a recent case-crossover study (an epidemiologic study design in which each case serves as its own control, allowing comparison of exposure at the time of disease onset to exposure at another time point) of 16,298 WNV cases in the United States showed that a 5°C increase in mean maximum weekly temperature is associated with a 32%–50% increase in WNV incidence (*4*). The first positive mosquito pool in the southern Okanagan Valley was detected ≈1 week after heavy rainfall and immediately after a period of extreme heat during which nightly temperatures were well above the 14.3°C limit for virus replication in *Cx. tarsalis* mosquitoes (*3*) (Figure 3). This rainfall likely increased the number of vector development sites; the ensuing period of high temperatures facilitated rapid mosquito development, virus amplification, and subsequent transmission in avian and mosquito populations.

The above-average abundance of *Cx*. *tarsalis* mosquitoes observed in 2009 is likely another key driver of the observed WNV range expansion (Table 1). *Cx*. *tarsalis* mosquitoes are bridge vectors that feed on birds and mammals (*33*). The elevated abundance of this species in 2009, especially the large peaks observed at the end of July and beginning of August (Figure 2), likely facilitated virus transmission from avian populations into humans and horses. However, *Cx. tarsalis* mosquitoes are much less common in British Columbia compared with other areas of Canada that experience large WNV outbreaks (*8**,**9*); this rarity may be 1 factor that has prevented past WNV activity in this region. Little is currently known about the ecology of *Cx*. *tarsalis* mosquitoes in the southern Okanagan Valley, and specific information is needed regarding the habitat preferences and overwintering practices of this species to enable more focused prevention efforts.

The detection of WNV in British Columbia in 2009 proves that the southern portion of the province contains the prerequisite environmental and ecologic conditions for within-season WNV amplification and transmission, at least in some years. What is less certain is whether the observed range expansions along the virus’s northern limit will lead to yearly endemic activity or to rare instances of sporadic disease as is typical in Washington State. WNV can overwinter in adult mosquitoes (*34*), thereby increasing the probability of future virus transmission in areas that had positive WNV indicators in 2009. Historic trends in some areas of the United States show marked increases in outbreak severity in the year after WNV introduction (*32*). Furthermore, the presence of a WNV-positive horse in the Fraser Valley is concerning given its proximity to British Columbia’s populated urban areas where *Cx*. *pipiens* mosquitoes are a consistent presence between June and August. Urban transmission of WNV in British Columbia in 2010 could lead to an increase in human cases and identifies a need for continued surveillance programs and appropriate prevention.

Outbreaks in human populations do require specific sequential weather conditions that may not be met in 2010 despite predictions for an El Niño year (*35*). In addition, the historically low abundance of key WNV vectors in British Columbia may limit WNV transmission in this region, and a return to provincial norms for *Cx*. *tarsali*s mosquito abundance could disrupt WNV transmission in the rural areas of the southern Okanagan Valley. St. Louis encephalitis virus, an arbovirus that shares vectors and reservoirs with WNV, was detected in southern British Columbia in mammals and humans in 1968 (*36*). Yet St. Louis encephalitis virus has caused no locally acquired human cases since, which indicates that arboviruses can circulate endemically in animal populations in the area without resulting in human cases.

In summary, the introduction and within-season amplification of WNV in 2009 represent a long-delayed range expansion. Although reasons for the delay remain unknown, we hypothesize that WNV activity in Washington State in 2009 provided, for the first time, a sufficient nearby source of WNV for northward introduction of the virus into British Columbia through cross-border mountain valleys. This introduction likely combined with uniquely warm nightly temperatures and elevated numbers of *Cx*. *tarsalis* mosquitoes in the southern Okanagan Valley; this combination of factors presented a convergence of favorable events that facilitated establishment and amplification in mosquito and avian populations. WNV activity levels in British Columbia in 2010 will provide valuable insight into the nature of WNV expansion and transmission along British Columbia’s northern and western borders. The presence of WNV activity in 2010, despite a return to normal temperatures and vector abundance, would suggest that ineffective virus introduction may be responsible for the prolonged absence of WNV in the province. Conversely, a return to normal temperatures and vector abundance combined with a lack of WNV activity in 2010 would suggest that environmental and ecologic conditions in this part of the Pacific Northwest are typically unsuitable for yearly WNV establishment, amplification, and transmission. Regardless, surveillance and ongoing consideration of appropriate prevention strategies are required to lessen the possibility of future WNV transmission to human populations in the region.
